# Value of rapid on‐site evaluation combined with interventional pulmonology techniques in the diagnosis of pulmonary cryptococcosis

**DOI:** 10.1111/crj.13746

**Published:** 2024-03-26

**Authors:** Jiaqi Cao, Rong Zhou, Qian He, Ming Zhang, Chunlai Feng

**Affiliations:** ^1^ Department of Respiratory and Critical Care Medicine The Third Affiliated Hospital of Soochow University Changzhou China

**Keywords:** interventional pulmonology techniques, percutaneous needle biopsy, pulmonary cryptococcosis, radial endobronchial ultrasound, rapid on‐site evaluation, transbronchial lung biopsy

## Abstract

**Objectives:**

The aim of this study is to evaluate the diagnostic value of rapid on‐site evaluation (ROSE) combined with computed tomography‐guided percutaneous needle biopsy (CT‐PNB) or radial endobronchial ultrasound‐guided transbronchial lung biopsy (EBUS‐TBLB) for pulmonary cryptococcosis (PC).

**Methods:**

Clinical data of 33 patients diagnosed with PC at the Third Affiliated Hospital of Soochow University between February 2018 and June 2023 were retrospectively analysed. Patients were divided into the CT‐PNB and EBUS‐TBLB groups based on the intervention method, and the diagnostic positivity rate and incidence of complications were compared between the two groups.

**Results:**

Compared with the final diagnosis, the positive diagnostic rates of ROSE, histopathology and serum CrAg of all patients were 81.8% (27/33), 72.7% (24/33) and 63.6% (21/33), respectively. The average turnaround times of the three methods were 0.1 (0.1–0.2) h, 96.0 (48.0–120.0) h and 7.8 (4.5–13.6) h, respectively (*P <* 0.001). The coincidence rate between histopathology and ROSE was 84.8% with a kappa value of 0.574. The positive diagnostic rate for PC was significantly higher in the CT‐PNB group than in the EBUS‐TBLB group (92.9% vs. 57.9%), and the difference was statistically significant (*P <* 0.05). Combined with the ROSE results, the positive diagnostic rate in the EBUS‐TBLB group increased to 84.2% (16/19).

**Conclusion:**

ROSE has commendable accuracy and timeliness, and CT‐PNB offers further advantages in this regard. ROSE enhances the diagnostic efficiency of EBUS‐TBLB for PC and is safe and effective.

## Graphical abstract

We analysed the diagnostic value and safety of rapid on‐site evaluation (ROSE) combined with computed tomography‐guided percutaneous needle biopsy (CT‐PNB) or radial endobronchial ultrasound‐guided transbronchial lung biopsy (EBUS‐TBLB) for pulmonary cryptococcosis (PC). We reveal that ROSE has commendable accuracy and timeliness, and CT‐PNB offers further advantages in this regard. We demonstrate that ROSE enhances the diagnostic efficiency of EBUS‐TBLB for PC and is safe and effective.

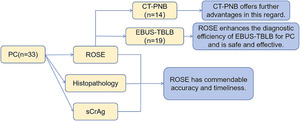

**Abbreviations**
CrAg: cryptococcal antigen
CT‐PNB: computed tomography‐guided percutaneous needle biopsy
EBUS‐TBLB: endobronchial ultrasound‐guided transbronchial lung biopsy
PC: pulmonary cryptococcosis
ROSE: rapid on‐site evaluation
TAT: average turnaround time

## 1 INTRODUCTION

Pulmonary cryptococcosis (PC), a subacute or chronic visceral fungal disease, is caused by inhalation of Cryptococcus spores or yeast cells through the respiratory tract.^1^ It mainly occurs in immunocompromised individuals, such as those with Human Immunodeficiency Virus (HIV) infections, organ transplant recipients and individuals undergoing corticosteroid or immunosuppressive therapy. However, its incidence has recently increased among immunocompetent individuals.^2^

The clinical manifestations and radiographic changes in PC lack distinct specificity. Its diagnosis relies mainly on pathogen smears or cultures, cryptococcal antigen (CrAg) detection and histopathology.^3^ Pathogen examination typically involves the collection of sputum or bronchoalveolar lavage fluid samples from patients for smear or culture; however, the positivity rate is low, and the process is time‐consuming. The serum CrAg test has high diagnostic sensitivity and specificity in patients with HIV and cryptococcal disease. However, its diagnostic sensitivity is low in non‐HIV patients with lung‐limited lesions. Histopathological results serve as the gold standard for PC diagnosis. In clinical practice, samples are primarily obtained through two interventional pulmonology techniques: computed tomography‐guided percutaneous needle biopsy (CT‐PNB) and endobronchial ultrasound‐guided transbronchial lung biopsy (EBUS‐TBLB). However, few studies have compared the diagnostic rates of these two methods for PC.

Rapid on‐site evaluation (ROSE) is a cytological technique that involves real‐time sampling.^4^ ROSE for percutaneous pulmonary puncture biopsy or transbronchial biopsy can enhance the diagnostic rate of pulmonary cancer, pulmonary nodules and peripheral pulmonary lesions and reduce the occurrence of secondary biopsies and complications.^5^, ^6^, ^7^, ^8^

However, few studies have investigated ROSE combined with interventional pulmonology techniques for PC diagnosis. This study aimed to analyse the diagnostic value and safety of ROSE combined with different interventional pulmonology techniques for PC.

## 2 METHODS

### 2.1 Subjects

In this retrospective analysis, 33 patients ultimately diagnosed with PC through histopathology or serum CrAg testing at the Third Affiliated Hospital of Soochow University between February 2018 and June 2023 were included.^9^

The inclusion criteria were as follows: (1) hospitalised patients with no gender limitation and ≥16 years of age, (2) not infected with HIV, (3) tissue specimens were obtained through CT‐PNB or EBUS‐TBLB, (4) PC diagnosed by histopathology or serum CrAg and (5) basic information were complete.

The exclusion criteria were as follows: (1) HIV infection, (2) those who did not undergo respiratory intervention and (3) those with incomplete basic information.

The study was approved by the Ethics Committee of our hospital, and all patients signed an informed consent form (Ethics 2019 no. 17).

### 2.2 Serum CrAg test

The CrAg lateral flow assay (CR2003, IMMY Inc, Oklahoma, America) helped identify CrAg presence in the serum according to the manufacturer guidelines.

### 2.3 CT‐PNB methods

**FIGURE 1:**
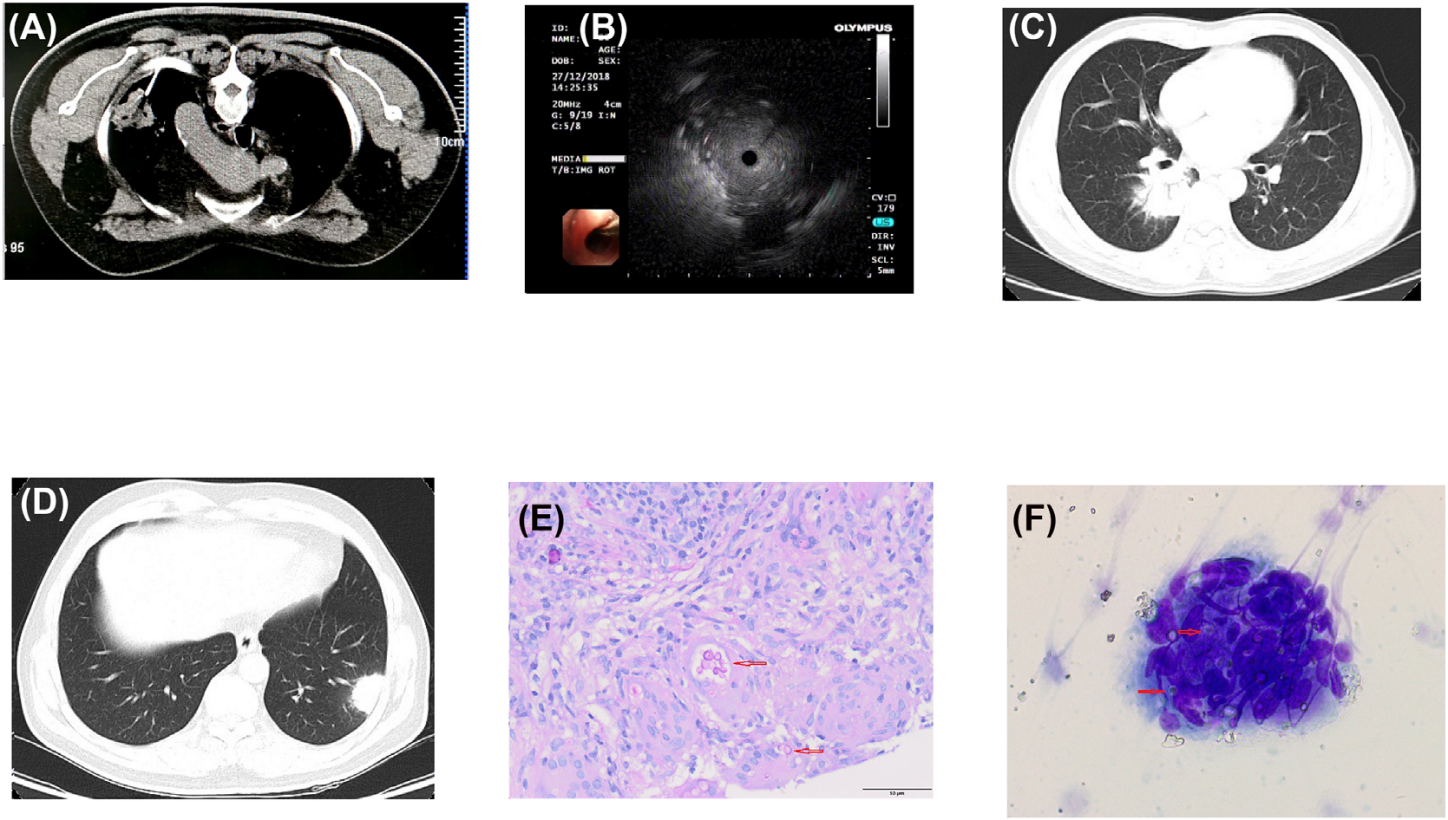
Image of CT‐PNB; (B) image of EBUS; (C, D) chest CT images in PC patients; (E) periodic acid–Schiff staining of tissue pathology in PC patient (red arrow, magnification ×400); (F) staining of rapid on‐site evaluation of cryptococcosis (red arrow, magnification ×400). CT, computed tomography; CT‐PNB, computed tomography‐guided percutaneous needle biopsy; EBUS, endobronchial ultrasound; PC, pulmonary cryptococcosis.

Before the intervention, all patients completed a chest CT scan with contrast to determine the vascular conditions surrounding the lesion and the position and puncture point according to the location of the lesion. The puncture process involved several steps. First, the patients underwent chest CT to obtain images that helped plan the optimal puncture points and paths. Once the puncture point was identified, marked and locally disinfected, 2% lidocaine was administered for local infiltration anaesthesia. The patients were then instructed to hold their breath, and a coaxial introducer needle (MCXS1815BP, Argon, Shanghai, China) was inserted at the designated puncture point, puncturing along the predetermined puncture angle; a subsequent chest CT scan was conducted to confirm that the puncture needle had reached the lesion. The needle core was then extracted, and a biopsy needle was inserted (360‐1580‐01, Argon) into the puncture needle (Figure 1A). The lung lesion tissue was swiftly cut one to three times. Once an ideal tissue was obtained, the biopsy needle and puncture needle were withdrawn. After local compression and disinfection, the puncture point was covered with sterile gauze. The diseased tissue was placed in a 4% formaldehyde solution for subsequent pathological examination. Following surgery, a chest CT scan was performed to rule out any bleeding or pneumothorax.

### 2.4 EBUS‐TBLB methods

A fiberoptic bronchoscope (BF‐P260F, Olympus, Tokyo, Japan), processor monitor (EU‐ME1; Olympus), ultrasonic host (MAJ‐935, Olympus) and R‐EBUS with a 1.4‐mm diameter (UM‐S20‐17S, Olympus) were used for R‐EBUS. A guiding sheath had (diameter 1.95 mm), and biopsy forceps (diameter 1.5 mm) were used (K‐201, Olympus). Before the interventional surgery, the lesion location was determined using preoperative thin‐layer CT under local infiltration anaesthesia combined with intravenous anaesthesia. A fasting period of 6 h for solid and liquid food was required before the surgery. The heart rate, blood pressure and pulse oxygen levels were monitored throughout the operation, with nasal oxygen provided as necessary to maintain blood oxygen saturation above 90%. During the surgery, the bronchoscope was first delivered to the bronchial lesion site based on CT imaging, and a small ultrasonic probe was inserted into the guide sheath to locate and fix the probe. The visible bronchial segments were continuously examined until a characteristic ultrasound signal indicating the presence of solid lesions was observed (Figure 1B). Subsequently, the EBUS probe was removed, and the sampling instrument was inserted through the guide sheath to obtain tissue samples.

### 2.5 ROSE procedures

The specimen was acquired by puncturing or forceping, fixed in Diff Quick A solution (DiffQuik; Baso Ltd., Guangzhou, Guangdong, China), stained for 30 s, slowly soaked in phosphate buffer, dried gently and then stained with Diff Quick B solution (Diff‐Quik; Baso Ltd.) for 40 s. Preliminary diagnostic information was obtained by observing cell composition and morphology under a microscope by an experienced pulmonologist. On successfully obtaining the sample, preliminary diagnosis was performed, tissue sampling was halted and the sample was sent for conventional analyses. Otherwise, the operation was repeated by fine‐tuning the angle of the needle for sampling at points adjacent to the lesion or adjusting the position and angle of the bronchoscope to ensure the timely acquisition of new sample.

### 2.6 Statistical analysis

Statistical analyses were performed using the IBM SPSS 26 software (IBM, Armonk, NY, USA). For normally distributed measurement data, the mean and standard deviation (Mean ± SD) were used. For measurement data that did not follow a normal distribution, the data were represented as M (P25‐P75), and multiple groups were compared using Kruskal–Wallis *H*‐test. Categorical variables are described as percentages and frequencies; Fisher's exact test was used to compare the data between the two groups. The Kappa value was calculated to evaluate consistency, and statistical significance was set at *P <* 0.05.

## 3 RESULTS

### 3.1 Basic information

This study included 33 patients. Their demographic characteristics, clinical symptoms, underlying diseases and chest CT findings are presented in Table 1.

**TABLE 1:** Basic information of patients with pulmonary cryptococcosis (*n* = 33).

Project	Patients
Age (years) (mean ± SD)	52.7 ± 14.8
Gender (*n* [%])	
Male	17 (51.5)
Female	16 (48.5)
Clinical symptoms (*n* (%])	
Fever	4 (12.1)
Cough/sputum	25 (75.8)
Dyspnoea	2 (6.1)
Chest pain	7 (21.2)
Haemoptysis	1 (3.0)
Asymptomatic	4 (12.1)
Underline illness (*n* (%])	
Diabetes	7 (21.2)
Solid organ malignancy	3 (9.1)
Myasthenia gravis	1 (3.0)
Rheumatic diseases	3 (9.1)
Other diseases	7 (21.2)
No underline diseases	12 (36.4)
Lumbar puncture	8 (24.2)
Radiographic presentation	
Lesion location (*n* (%])	
Right upper lobe	10 (30.3)
Right middle lobe	6 (18.2)
Right lower lobe	20 (60.6)
Left superior lobe	7 (21.2)
Left lower lobe	16 (48.5)
Lesion characteristics (*n* (%])	
Single or multiple nodular massive lesions	17 (51.5)
Lamellar infiltration	13 (39.4)
Diffuse mixed lesions	3 (9.1)

### 3.2 Histopathology and staining

Histopathology revealed capsulated cryptococcal bodies, spores or granulomatous inflammation of multinucleated giant cells, epithelioid cells and lymphocytes with or without necrosis. Positive staining with Grocott methenamine silver or Periodic acid–Schiff indicated the likelihood of PC (Figure 1E).

### 3.3 ROSE performance

ROSE smear showed multinucleated giant cells formed from epithelial‐like cells. Circular mycelium (diameter 4–6 μm) was visible in the multinucleated giant cell cytoplasm. A transparent and thick capsule on the outside, with deeply stained irregular spores inside, with more activated lymphocytes were observed. This indicated cryptococcal infection (Figure 1F).

### 3.4 Comparing results of ROSE and Histopathology

The coincidence rate of ROSE and histopathological diagnosis was 84.8%, and the kappa value was 0.574 (*P <* 0.01), indicating good consistency between the two methods (Table 2).

**TABLE 2:** Comparing results of ROSE and histopathology.

ROSE	Histopathology		Total
	Positive	Negative	
Positive	23	4	27
Negative	1	5	6
Total	24	9	33

Abbreviation: ROSE, rapid on‐site evaluation.

### 3.5 Diagnostic positive rate analysis

ROSE, histopathology and serum CrAg tests were performed for all 33 patients. Among them, 27 were diagnosed with ROSE, 24 with histopathological findings and 21 with CrAg. The positivity rates of the three methods were 81.8% (27/33), 72.7% (24/33) and 63.6% (21/33), respectively.

Tissue specimens were obtained from 19 patients using EBUS‐TBLB and 14 patients using CT‐PNB. Patients were divided into EBUS‐TBLB and CT‐PNB groups according to different interventional pulmonology techniques.

Among the 19 patients in the EBUS‐TBLB group, 11 were confirmed by histopathology, and the positivity rate was 57.9% (11/19); 15 cases were diagnosed by ROSE, and 12 were diagnosed by the serum CrAg. When combined with ROSE, the positivity rate increased to 84.2% (16/19). The combination of ROSE and serum CrAg increased the diagnostic positivity rate to 94.7% (18/19).

Among the 14 patients in the CT‐PNB group, 13 were confirmed by histopathology, and the positive rate was 92.9% (13/14); 12 were diagnosed with ROSE, and 9 were diagnosed with serum CrAg. The combination of ROSE and serum CrAg increased the positivity rate to 100% (14/14).

### 3.6 Comparing the positive rate of diagnosis and complications between the two groups

The positive diagnostic rates in the EBUS‐TBLB and CT‐PNB groups were 57.9% and 92.9%, respectively, with statistically significant differences (*P* < 0.05). No pneumothorax or haemoptysis was observed in the EBUS‐TBLB group. In the CT‐PNB group, the incidence of pneumothorax was 21.4% (3/14), and the incidence of haemoptysis was 28.5% (4/14); the difference in the incidence of pneumothorax between the two groups was not significant (*P* > 0.05), although the incidence of haemoptysis was statistically significant (*P* < 0.05) (Table 3).

**TABLE 3:** Comparison of diagnosis positive rate, complications and CT imaging features between the two groups.

Project	EBUS‐TBLB	CT‐PNB	*P‐*value
Positive diagnostic rate (%)	57.9	92.9	0.047
Pneumothorax incidence rate (%)	0	21.4	0.067
Incidence of haemoptysis (%)	0	28.5	0.024
Radiographic feature			
Lesion located in outside 1/3 band (*n* (%])	5(26.3)	13(92.9)	<0.001
Attached to the chest wall (*n* (%])	1(5.3)	9(64.3)	<0.001
Positive bronchial sign (*n* (%])	16(84.2)	3(21.4)	<0.001

Abbreviations: CT‐PNB, computed tomography‐guided percutaneous needle biopsy; EBUS‐TBLB, endobronchial ultrasound‐guided transbronchial lung biopsy.

The pulmonary radiological characteristics of the two groups are presented in Table 3. Differences in the three imaging features between the two groups were statistically significant. In addition, 16 patients in the EBUS‐TBLB group had bronchial signs (Figure 1C), of which 11 (68.7%) were pathologically positive; the positive pathological diagnosis rate of the three patients with negative bronchial signs was zero. In the CT‐PNB group, the lesions were located in the outer 1/3 band (Figure 1D), and the pathological diagnosis was 100% positive.

### 3.7 Comparison of timeliness

The average turnaround time (TAT) required for ROSE, histopathology and serum CrAg from obtaining tissue or blood samples to issuing a formal report to assist in the diagnosis of PC were 0.1 (0.1–0.2) h, 96.0 (48.0–120.0) h and 7.8 (4.5–13.6) h, respectively (*P <* 0.001).

## 4 DISCUSSION

Variations exist in the clinical manifestations and imaging findings of PC depending on the immune status of individuals. Immunocompetent patients often have no clinical or mild symptoms^10^, ^11^; most lung lesions are limited, with nodular mass types being the most common.^12^ Immunocompromised patients present with a high fever and acute respiratory failure.^13^ HIV‐negative immunosuppressed patients have pneumonia‐type commonly observed lesions,^12^ whereas HIV patients often exhibit diffuse interstitial changes.^14^ Herein, the study participants were HIV‐negative, predominantly with normal immune function that presented with cough as their primary symptom and rarely high fever. CT tomography revealed nodules, masses or limited flaky shadows as the most prevalent findings. Owing to the nonspecific clinical characteristics of these patients, they are susceptible to misdiagnosis of lung cancer, pulmonary tuberculosis, pneumonia and other diseases; therefore, timely and accurate diagnosis is crucial.

Histopathology has a high diagnostic value for PC; however, it is time‐consuming rendering it unsuitable for early diagnosis and timely treatment.^15^, ^16^ The expert consensus on invasive fungal diseases recognises cytopathological results as a crucial basis for PC.^9^ ROSE, a cytological technique, can not only be used for diagnosing PC but also has advantages in terms of timeliness. This study compared the average TAT required for ROSE, histopathological examination and serum CrAg. ROSE required the shortest time. The ROSE result was consistent with the histopathological results for pulmonary cancer and peripheral pulmonary lesion doagnosis.^8^, ^17^, ^18^ A high coincidence rate between ROSE and histopathological findings, with a kappa value of 0.574, was also observed; the positive diagnostic rate of PC did not change significantly. Therefore, ROSE has good consistency with histopathology for PC diagnosis and exhibits a certain level of accuracy and timeliness.

While clinically diagnosing pulmonary diseases, interventional methods or surgeries are typically used to obtain tissue samples. Compared with surgery, interventional pulmonology techniques have the advantages of less trauma, lower incidence and milder severity of complications. ROSE can guide through the operation in real time at the clinical operation site and preliminarily determine the adequacy and qualification rate of specimens. Its use during respiratory intervention procedures effectively enhances the positive diagnostic rate.^19^, ^20^ In this study, all included patients were sampled using EBUS‐TBLB or CT‐PNB, and ROSE was performed during the sampling process, achieving a high diagnostic positivity rate. It may be related to the close combination of ROSE results by clinical doctors during the intervention process and evaluating the adequacy of the biopsy specimens. The positive diagnostic rate of PC in the CT‐PNB group was significantly higher than that in the EBUS‐TBLB group. Further analysis of the radiographic characteristics of these patients revealed that, in the CT‐PNB group, 92.9% of the lung lesions were located in the outer 1/3 band and 64.3% of the lesions were close to the chest wall. These factors were advantageous for successful percutaneous lung puncture and acquisition of sufficient tissue for histopathological examination. Therefore, for patients suspected of PC, when the lesion is located in the outer lung or near the pleura as per chest CT, CT‐PNB can be given priority to improve the positive diagnostic rate.

Positive bronchial signs were favourable factors for the diagnostic rate of transbronchoscopic biopsy.^21^ In this study, patients in the EBUS‐TBLB group mainly had lesions located in the inner and middle bands and 84.2% had bronchial signs. The pathological diagnosis positivity rate in the bronchial sign‐positive group was 68.7% higher than that in the bronchial sign‐negative group, although the difference was not significant. Considering that this may be related to the small sample size, samples can be accumulated for further studies in later stages.

The diagnostic value of a transbronchial biopsy partly depends on the size of the biopsy specimen. For infectious diseases, the larger the size and more the number of biopsy specimens, the higher the efficiency of pathological diagnosis.^22^ Compared with CT‐PNB, EBUS‐TBLB acquires a smaller tissue size, is less traumatic and can be performed multiple times. ROSE can guide the bronchoscopy process in real time, initially determine the adequacy and qualification rate of specimens and guide further auxiliary examinations, including microbial culture and antigen detection, which helps increase the positive diagnostic rate of infectious diseases.^23^, ^24^ In this study, EBUS‐TBLB combined with ROSE significantly improved the diagnostic positivity rate and on this basis, when combined with CrAg, achieved the same diagnostic results as CT‐PNB. ROSE can evaluate the quality of the sample during bronchoscopy, distinguish the cytological manifestations of cryptococcal infection and the structural characteristics of the pathogen in the specimen in time and help guide the doctor to send the lavage fluid or blood sample for CrAg testing to improve the diagnosis rate of cryptococcal disease.

Furthermore, EBUS‐TBLB has the advantage of fewer complications than CT‐PNB. In summary, both EBUS‐TBLB and CT‐PNB have advantages and disadvantages, and it is important to comprehensively consider the patients' basic conditions and radiographic features.

This study has limitations. The sample size was small, and future research aiming to accumulate more cases and analyse factors that exhibit differences but lack statistical significance in the current study is needed. Secondly, this study was a retrospective, nonrandomised, single‐centre study and lacked standardised control analysis, which may have introduced selection bias. Therefore, the diagnostic value of ROSE combined with respiratory intervention methods for PC requires further confirmation using larger sample sizes and prospective multicentre cohort studies.

## 5 CONCLUSIONS

ROSE aligns well with histopathological examination in diagnosing PC and offers timely results. The combination of ROSE and respiratory intervention methods enhances the diagnostic efficiency of PC, and its application in clinical practice is worth promoting.

## AUTHOR CONTRIBUTIONS

The concept and design of this study were described by Feng et al. The data were acquired, analysed and interpreted by Jiaqi Cao, Qian He and Ming Zhang. The manuscript was drafted by Jiaqi Cao and Rong Zhou. Chunlai Feng critically revised the manuscript for intellectual content. All the authors have read and approved the final manuscript.

## CONFLICT OF INTEREST STATEMENT

None.

## ETHIC STATEMENTS

The authors declare that the study received ethical approval from the relevant clinical research ethics committee and conformed to the Code of Ethics of the World Medical Association (Declaration of Helsinki).

## RIGHT TO PRIVACY AND INFORMED CONSENT

Written informed consent was obtained from all patients included in this study. The corresponding author holds the document for publication.

## Data Availability

The data supporting the findings of this study can be obtained from the corresponding author upon request.
